# Women’s country of birth and failure to catch up an overdue cervical cancer cytological screening participation during pregnancy in France, an observational study based on survey sources

**DOI:** 10.1186/s12885-024-12335-1

**Published:** 2024-05-16

**Authors:** Elisabeth Lyonnais, Solène Vigoureux, Béatrice Blondel, Sophie Wylomanski, Elie Azria

**Affiliations:** 1 Obstetrical, Perinatal and Pediatric Epidemiology (EPOPé Research Team), FHU Prema, Université Paris Cité – INSERM, 75014 Paris, France; 2 Maternity Unit, Paris Saint Joseph Hospital, Paris, France

**Keywords:** Cervical cancer screening, Pap test, Pregnancy, National perinatal survey, Health inequalities

## Abstract

**Background:**

Cervical cancer is the fourth most common cancer among women worldwide, both for incidence and mortality. Prevention relies on screening with a Pap test to detect precancerous lesions, which can then be treated. Access to this screening is currently both improvable and inequitable. Pregnancy may be an ideal moment for women to catch up on their overdue cervical cancer screening. In the general population, women's risk of not being screened is associated with their place of birth and other social factors; this may be true as well among pregnant women. Our objective was to study the association between women's place of birth and their failure to catch up with this screening during pregnancy.

**Methods:**

The 2016 French National Perinatal Survey included 13,147 women who gave birth after 21 weeks of gestation. The association between their place of birth and failure to catch up on this screening (defined by the absence of a Pap test during pregnancy for women overdue for it) was adjusted for age, parity, education level, health insurance, and when they began prenatal care with logistic regression models.

**Results:**

Among the women for whom screening was then recommended, 49% were not up to date at the start of pregnancy, and of these, 53% were not caught up before delivery. After adjustment for other risk factors, maternal place of birth was not associated with a higher risk of failure to catch up with this screening during pregnancy. However, factors identified as associated with this risk included a low education level and late start of prenatal care.

**Conclusion:**

About half of women overdue for cervical cancer screening did not catch up with it during their pregnancy. Professionals should pay special attention to women with lower education levels and late initiation of prenatal care, who constitute a group at high risk of not catching up on this screening during pregnancy.

## Background

The World Health Organization (WHO) reports that the incidence of cervical cancer was 660,000 worldwide in 2022 and that more than 350,000 women died from it that year [1].

Prevention relies on screening with a Pap test to detect precancerous lesions that can then be treated. In Europe, screening and treating early neoplasia have substantially reduced the incidence and mortality of cervical cancer since 1960 [2–5]. In this area in 2021, rates of women aged 30–49 who reported ever having had a cervical cancer test varied from 42.0% (in Romania) to 98.4% in Finland, according to the WHO [6].In other high-income countries, rates were higher: 88% in the USA, 91% in Canada, and 95% in Australia had ever undergo Pap tests [7].

From 2010 to 2019, French guidelines recommended that women have a Pap test every 3 years between the ages of 25 and 65 years, after they have had two normal Pap smear results one year apart [8]. These guidelines, however, have been poorly implemented in France: only 58.7% of women were screened every three years between 2015 and 2017 in France [9].

Low participation rates in screening programs increases the risk of dying from invasive cervical cancer, and every year in France, 60% to 70% of the new cases of this cancer are diagnosed among women aged from 35 to 69 years who are unscreened or underscreened [8]. Recent studies show that risk factors for such non-screening or underscreening include a low education level and/or low income, living alone, unemployment, and lack of medical insurance, compared with women living in more privileged environments [10–21]. Some studies have also found that migrant women are screened less often than native women [11, 22–27]. In Canada, migrant women have an adjusted RR 1.32; 95% CI 1.20–1.45) for an overdue Pap test compared to Canadian-born women [25]. Several French studies found that foreign women born to foreign parents underwent recommended cervical cancer screening less often than French women born to foreign parents, who themselves were less likely to be screened than French women born to French parents [12, 28, 29]. In Norway, Enden and al. showed that, despite a global increase of cervical cancer screening participation between 2012 et 2017, this increase was significantly smaller among immigrant women compared to Norwegian-born women [24].

Because pregnancy is a privileged moment for access to health care, it might be a good time to catch up with gynecologic follow-up for women not receiving regular triennial screening [8].

Although the performance of this screening has not been evaluated in pregnant women, the French Health Authority has recommended since 2007 Pap tests for all woman at the beginning of pregnancy if their last test took place more than three years earlier [30]. Maternal health inequalities according to maternal place of birth have been described in high-income countries, specifically in France [31, 32]. It is important to know if these inequalities also affect cervical cancer screening during pregnancy.

The objectives of this study were to describe the association between mothers' place of birth and their failure to catch up on cervical cancer screening during pregnancy and to identify whether some other social characteristics might be risk factors for this among a national sample of women giving birth in France.

## Methods

### Data sources

The study population came from the French National Perinatal Survey conducted in March 2016. These surveys are fairly regular population-based cross-sectional studies using the same methodology and including all births (live births and stillbirths) after 21 weeks’ gestation or with a birthweight of at least 500 g during a one-week period in all maternity units in France [33].

For each birth, data were collected by a face-to-face interview and the collection of information from the medical records by a midwife. Maternal socioeconomic characteristics and prenatal care were obtained during the interview. Each woman was asked about a Pap test during pregnancy and over the past three years.

**Fig. 1:**
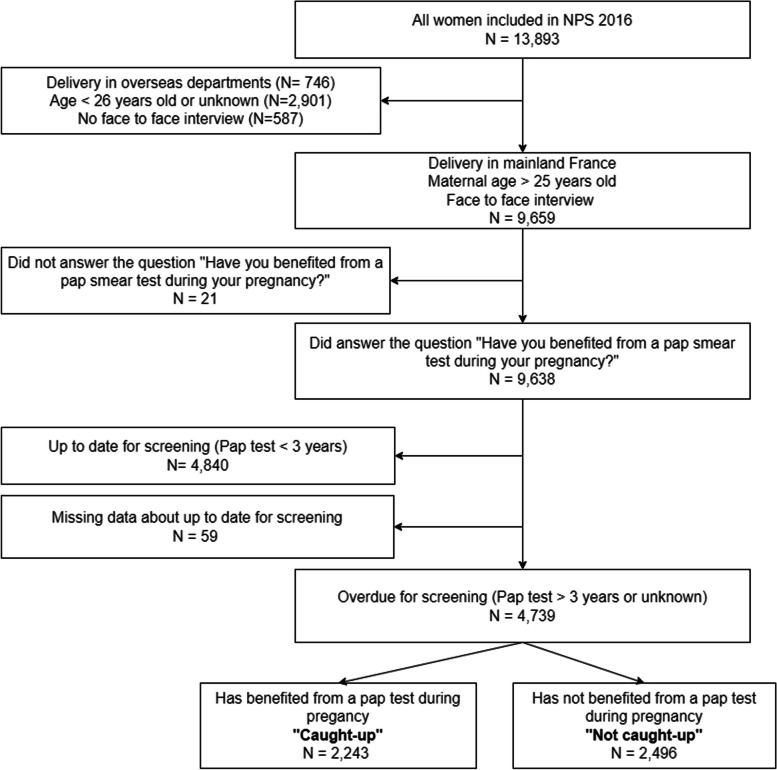
Study population

The perinatal survey database included 13,893 women (Fig. 1 Study population). The study population included all women who gave birth in mainland France, were more than 25 years old, and were interviewed and answered the question about a Pap test during pregnancy and over the past 3 years.

### Variables

#### Outcome measurement

The outcome was the performance of a Pap test during pregnancy for women aged 26 years or older who were not up to date for this screening.

We considered women to be “up to date” for screening when they reported having had a Pap test in the previous three years. Women were considered overdue for it when they reported that their last Pap test took place more than three years earlier or that its date (if any) was unknown.

Among the overdue women, those who answered “Yes” to the question about a Pap test during pregnancy are classified as “caught up” and those who answered “No” or did not remember having had a Pap test during pregnancy were considered as “not caught up”.

#### Exposure measurement

Maternal place of birth was classified in five categories: France, other European countries, North Africa, other African countries, and elsewhere in the world.

#### Social and demographic characteristics

- Maternal age was divided into three categories: 26–30 years old, 31–35 years old, or 36 years older or more.
- Education level was the highest level of education, again in three categories: Middle school or less, high school and beyond high school.
- Socioeconomic situation was defined by several characteristics:
- Employment status during pregnancy: Employed, unemployed and/or looking for work, homemaker or student or other.
- Personal housing during the last trimester of pregnancy, as a binary variable: yes or no.
- No work-related household income, as a binary variable: yes or no.
- Standard health insurance coverage at the beginning of pregnancy, as a binary variable: yes or no.
- Living with a partner as a binary variable: yes or no.

#### Inadequate prenatal care utilization

We used the indicator described by Gonthier et al. [34] to assess the adherence of prenatal care to current French recommendations. It covers late initiation of care (started later than 12 weeks of gestation) and too few appointments (i.e. < 7 prenatal visits and 3 ultrasound examinations for full term pregnancies) and is defined specifically as:

- Late initiation of care

And/or

- Fewer than half the number of prenatal visits expected according to the duration of pregnancy

And/or

- Insufficient number of ultrasound screenings: missing either the first-trimester ultrasound examination or both the second- and third-trimester examinations

#### Statistical analyses

The population was described by comparing the women overdue at the start of pregnancy who were and were not "caught up" by its end for proportions of categorical variables. To study the association between each social factors and failure to catch up on screening, we performed a bivariate analysis. Then, we constructed a multivariate logistic regression model adjusted for maternal place of birth, age, parity, education level, health insurance coverage, and timing of prenatal care initiation. Associations between failure to catch up, mother’s place of birth, and covariates were expressed as crude odds ratios (OR) and adjusted odds ratios (AOR) and their 95% confidence intervals (CIs).

STATA 15.0 software was used to perform the analyses.

## Results

### Description

Women included in the National Perinatal Survey and who gave birth in mainland France, were more than 25 years old (eligible for cervical cancer screening), and answered the question about a Pap test during pregnancy and over the past 3 years were 9,638 (Fig. 1 Study population). Among the latter, 4,840 women reported they were up to date for cervical cancer screening because they had had a Pap test in the previous three years, while 4,739 women (49%) were overdue. Among these overdue women, 2,243 (47%) answered the question about a Pap test during pregnancy positively and are considered caught up, while 1,862 (53%) answered negatively or did not remember and were considered not caught up.

Not caught up women were younger (chi-square p test = 0.001), less well educated (*p* < 0.0001), and less often employed (*p* = 0.002) than women who were caught up (Table 1). They also initiated prenatal care later than caught-up women and had an inadequate prenatal care utilization more frequently (*p* < 0.0001).

**Table 1:** Description of the population of overdue women according to the catch-up during pregnancy

	Total	Caught up		Not caught up		*P*-value*
	*n* = 4,739	*n* = 2,243		*n* = 2,496		
	n	n	%	n	%	
Maternal age (n, %)						*0.001*
26–30 years	2,022	894	40	1,128	45	
31–35 years	1,808	912	41	896	36	
More than 35 years	909	437	19	472	19	
Parity (n, %)						0.14
Primiparous	1,652	758	34	894	36	
Multiparous	3,086	1,485	66	1,601	64	
Education level (n, %)						< *0.0001*
Middle school or less	1,100	474	21	626	25	
High school	992	437	20	555	22	
Beyond high school	2,603	1,304	59	1,299	52	
Maternal place of birth (n, %)						*0.45*
France	3,569	1,711	76	1,858	74	
Other European countries	219	103	5	116	5	
North Africa	456	197	9	259	10	
Other African countries	298	139	6	159	6	
Elsewhere in the world	197	93	4	104	4	
Employment status (n, %)						*0.002*
Employed	3,079	1,520	69	1,559	64	
Unemployed and/or looking for work	763	343	16	420	17	
Homemaker	675	292	13	383	16	
Student or other	111	43	2	68	3	
Single (n, %)						0.30
	271	120	5	151	6	
No work-related household income (n, %)						0.34
	458	207	9	251	10	
No standard health insurance coverage (n, %)						< *0.0001*
	744	307	14	437	18	
No personal housing (n, %)						< *0.0001*
	250	88	4	162	6	
Late initiation of prenatal care (n, %)						< *0.0001*
	148	41	2	107	4	
Inadequate prenatal care^a^ (n, %)						< *0.0001*
	290	97	4	193	8	

All values are expressed as n and percentage of overdue women

^*^p-value for chi-squared test

^a﻿^Late initiation of care and/or fewer than half the number of prenatal visits expected according to the duration of pregnancy and/or insufficient number of ultrasound screenings

### Factors associated with not catching up: bivariate and multivariate analyses

We did not observe with the bivariate analysis any association between maternal place of birth and failure to catch up during pregnancy (Table 2). The analysis however showed that several factors were associated with failing to catch up, including having non-standard (versus standard) health insurance at the beginning of pregnancy (Crude OR 1.34 95% CI [1.14–1.57]) and a middle school or high school education level (versus beyond high school level) (Crude OR 1.33 95% CI [1.15–1.53] and 1.27 95% CI [1.10–1.48] respectively).

**Table 2:** Factors associated with not catching up in the overdue population: bivariate and multivariate analysis

	No. of women	Not caught up women (%)	Crude OR	95% CI	p value*	No. of women	AOR	95% CI	*P* value*
Maternal place of birth	4,739				0.41	4,601			0.87
France	3,569	52	Ref	-			Ref	-	
Other European countries	219	53	1.04	[0.79,1.36]			1.01	[0.76,1.33]	
North Africa	456	57	1.21	[0.99,1.47]			1.07	[0.86,1.32]	
Other African countries	298	53	1.05	[0.83,1.33]			0.89	[0.69,1.16]	
Elsewhere in the world	197	53	1.03	[0.77,1.37]			1.00	[0.74,1.35]	
Age	4,739								*0.01*
26–30 years	2,022	56	Ref	-	*0.0005*		Ref	-	
31–35 years	1,808	50	0.78	[0.69,0.88]			0.82	[0.72,0.94]	
> 35 years	909	52	0.86	[0.73,1.00]			0.88	[0.74,1.03]	
Parity	4,738				0.14				0.08
Multiparous	3,086	52	Ref	-			Ref	-	
Primiparous	1,652	54	1.09	[0.97,1.23]			1.12	[0.98,1.27]	
Level of education	4,695				< *0.0001*				*0.006*
Beyond high school	2,603	50	Ref	-			Ref	-	
High school	992	56	1.27	[1.10,1.48]			1.21	[1.04,1.41]	
Middle school or less	1,100	57	1.33	[1.15,1.53]			1.24	[1.06,1.45]	
Standard health insurance coverage	4,735				*0.0001*				0.11
Yes	3,991	51	Ref	-			Ref	-	
No	744	59	1.34	[1.14,1.57]			1.16	[0.97,1.40]	
Late initiation of prenatal care	4,646				< *0.0001*				*0.0001*
No	4,498	52	Ref	-			Ref	-	
Yes	148	72	2.43	[1.69,3.50]			2.13	[1.46,3.10]	

*Crude OR* Crude Odd Ratio, *95% CI* 95% Confidence Interval, *AOR* Adjusted OR on maternal place of birth, age, parity, level of education, standard health insurance coverage and late initiation of prenatal care

^*^p value for chi-squared test

In the multivariate analysis, after adjustment for age, parity, level of education, health insurance and late initiation of prenatal care, the maternal place of birth was not significantly associated with the risk of not being caught up. On the other hand, having a middle school or high school education level was significantly associated with not catching up (AOR 1.24 95% CI [1.06–1.45] and AOR 1.21 95% CI [1.04–1.41] respectively), compared with women with higher qualifications. Late initiation of antenatal care was strongly associated with failure to catch up (AOR = 2.13 95% CI [1.46–3.10]).

The proportion of missing data was less than 2% for each variable. Observations containing missing data were excluded from the multivariate analysis which was performed on 4,601 complete observations out of 4,739.

## Discussion

This analysis of the 2016 French National Perinatal Survey shows that 49% of the women eligible for cervical screening were overdue for it, and among this group, 53% did not catch up with this screening during their pregnancy, despite national guidelines strongly recommending it. Maternal place of birth was not associated with this failure to catch up during pregnancy, although an age of 26–30 years, a lower education level, a start of prenatal care later, compared with overdue women who were caught up, were associated with it.

### Strengths and limitations

One of the strengths of this analysis is the large number of women included and the low rate of missing data; these factors together provide good statistical power and limit the risk of bias. The survey's design also ensures the sample's representativity. The participation of nearly every maternity unit in France resulted in a number of births very close to that expected according to the INSEE statistics; at the same time, the characteristics of the mothers, deliveries, and newborns were similar to those already known through hospital discharge summaries (PMSI) [33].

Nonetheless, women not speaking French well did not have face-to-face interviews and were thus excluded from this analysis. They accounted for almost 4% of the women aged 26 years or older. Most of them were immigrants and perhaps among the most deprived individuals in our sample. This selection bias might have led us to underestimate the strength of the associations between social factors and failure to catch up. On the other hand, excluding these women from the study and analysis might have prevented us from being able to highlight an existing association between immigration and catch-up failure.

Another limitation is related to the quality of the data collected about prenatal care. Women may have forgotten, omitted, or misunderstood some questions. They may confuse Pap tests with simple vaginal samples. A few studies suggested that women over report Pap tests, partly by equating any examination of the pelvic area to a Pap test [9, 35–37]. Women with a low level of education or with a language barrier may therefore have more often misunderstood this question; some women may not have been considered caught up although they had had a Pap test, or the inverse might be true.

While many authors have asked if social and economic status influences the rate of reporting the response is not unanimous: some authors find over-reporting among the most disadvantaged, others among the most advantaged, while still others find no association between social background and reporting [35, 36, 38]. Lastly, during the National Perinatal Survey, the interview was carried out by a midwife, who could help women remember this test and could have limited memorization bias.

### Interpretation of results

#### Failure to catch up

First, almost half of all pregnant women were overdue for cervical cancer screening in France in 2016, and slightly more than half did not catch up during pregnancy. French hospital-based studies have found similar rates of failure to catch up during pregnancy (from 53 to 61%) [39–41], but our work is the first study to describe this phenomenon among a national sample of pregnant women. In the UK, Coleridge et al. found that nearly half (47.3%) of a sample of 260 pregnant women were overdue for cervical screening and 74% were not caught up during either their pregnancy or the first 6 months postpartum [42]. In Brazil, Terlan and Cesar have observed that, despite prenatal visits, 21.6% pregnant women did not undergo the Pap smears they should have had [43].

Despite these inadequate catch-up rates during pregnancy, some countries have shown that this period does indeed present an important opportunity for health care professionals to help women to catch up with overdue screening. A Norwegian cohort study including more than 2 million women showed that pregnant women were almost five times more likely to have a Pap smear test within one year compared to the non-pregnant women [44]. A Polish hospital-based study found that 7.5% of women older than 25 years reported that the Pap test performed during pregnancy, in accordance with local guidelines, was the first they had ever had.

#### Maternal place of birth

In our analysis, maternal place of birth was not associated with failure to catch up with cervical cancer screening during pregnancy. To our knowledge, this study is the first to assess specifically the association between maternal place of birth and this screening during pregnancy. Moreover, we have not found studies that investigated the associations between maternal nationality or ethnicity and cervical cancer screening. Most studies concern associations between women’s place of birth or ethnicity in general populations.

Several Canadian studies have shown significant cervical cancer screening inequalities based on age, income, immigration status, and world region of origin [25, 27]. A review of the literature conducted in 2019 showed that women from sub-Saharan Africa and living in Canada origin had the lowest cervical cancer screening rates [45].

In Norway, women from North and sub-Saharan Africa had lower rates of participation in cervical cancer screening programs than Norwegian-born women (adjusted OR 0.61, 95% CI [0.56–0.67]) [46]. In Denmark, migrant women have the lowest rate of participation in the national screening program, even after adjustment for other social characteristics. The authors suggest that this result might be due to a language barrier, some difficulties in understanding the screening invitation (written in Danish), and poor health literacy — all barriers to seeking care or understanding and adhering to prevention and screening [11, 47]. According to Idehen et al., Russians, Somalis and Kurds women living in Finland are less screened than Finnish women [26].

In France, Sassenou et al. observed in 2023 that women residing in France and born in European countries other than France were screened less often than native women [29]. The lack of association between maternal place of birth and catch-up screening during pregnancy, analyzed in a selected population of overdue pregnant women, does not however reflect an association that would exist outside pregnancy between place of birth and access to cervical cancer screening.

#### Age

Age was also associated with failure to catch up. In our study, the youngest pregnant women had had fewer Pap tests than those older than 30 years. In the Polish study by Kusczborska et al., age was the only factor associated with Pap tests both before and during the current pregnancy, but it enrolled women younger than 25 years, who are normally not subject to Polish screening guidelines [48]. In Brazil, Monteiro et al. and Cesar et al. found that young age (younger than 35 years old, respectively) was associated with lower Pap testing rates during pregnancy [49, 50].

#### Adherence to medical guidelines

Late initiation of prenatal care was associated with failure to catch up on screening. This may be due to the care provider's concern about performing a Pap test after the first trimester and suggests poor knowledge of current guidelines. The French Health Authority guidelines, the French Public Health Code, and the guidelines of the French National College of Gynecologists and Obstetricians state that a Pap test can be performed at any time during pregnancy, especially for women without regular gynecological follow-up [40, 51]. Nonetheless, among a sample of French midwives interviewed in 2018, 29% reported that they would perform a Pap test at 25 weeks of gestation, compared with more than 90% at 10 weeks [52]. In a study that took place in 2009–2010 in a University Hospital Center of France, the proportion of adequate screening (defined by performing a Pap test during pregnancy if the last one was more than two years earlier or if its result was unknown) was significantly higher when the first prenatal visit occurred during the first trimester rather than during the second or the third trimester (48% versus 12%) [41]. According to Saulneron et al. most Pap tests performed during pregnancy take place during the first trimester (86.7%) [40]. In the Norwegian cohort of Nygard et al., most Pap smears from pregnant women were taken during the first 4 months of pregnancy [44].

A Pap test can also be proposed during the postnatal visit, but several studies have shown that 68% to 83% of women do not attend this visit, in particular, those in situations of social deprivation [53–55]. This non-adherence results in missing the opportunity for these women to be caught up with this important preventive care, in particular, those with a poor access to gynecological care [56, 57].

Strong public health policies could reduce the late initiation of prenatal care and thereby have a positive impact on cervical cancer screening during pregnancy. In Norway, the high rate of participation of pregnant women in the national screening program has improved its coverage throughout the female population [44].

In 2019, the French Health Authority (HAS) published new recommendations on cervical cancer screening, advising an HPV test every five years for women over 30, rather than Pap tests [58]. These new guidelines, if well disseminated to and adhered to by health care providers, may improve screening of pregnant woman overdue for cervical cancer screening.

Individual factors play a moderate role in failed catch up of women overdue for screening during pregnancy. A better understanding of why recommendations are so poorly implemented requires a study of the knowledge, attitudes, and practices of all health care providers.

## Conclusion

Despite guidelines, nearly half of all pregnant women are overdue for cervical cancer screening, and catch-up will not occur for 53% of them during pregnancy. A young age (younger than 30 years), a low education level, and late initiation of prenatal care are factors associated with failure to catch up, but maternal place of birth does not appear to be an independent risk factor. Health care professionals must be made aware of these factors, so that women who are overdue for screening, particularly those most at risk, can catch up. It is important that professionals involved in prenatal care understand the new screening procedures well and can implement them, even for women whose prenatal care begin late.

## Data Availability

A description of the study is available from: enp.inserm.fr. The data are partially accessible from the following link http://quetelet.progedo.fr/.
